# The path towards a separate residency in “Neonatal Critical Care Medicine” has been paved in Europe

**DOI:** 10.1038/s41372-026-02769-9

**Published:** 2026-06-22

**Authors:** C. C. Roehr, W. de Boode, T. Szczapa, O. Danhaive, M. Vento, A. Alarcon-Allen, P. Fentsch, B. Koletzko, S. Wellmann

**Affiliations:** 1 Faculty of Health and Life Sciences, University of Bristol, Bristol, UK; 2 Southmead Hospital, North Bristol NHS Trust, Bristol, UK; 3 National Perinatal Epidemiology Unit, Nuffield Department of Women’s and Reproductive Health, University of Oxford, Oxford, UK; 4 Department of Neonatology, Amalia Children’s Hospital, Radboud University Medical Center (Radboudumc), Nijmegen, The Netherlands; 5 II Department of Neonatology, Neonatal Biophysical Monitoring and Cardiopulmonary Therapies Research Unit, Poznań University of Medical Sciences (PUMS), Poznań, Poland; 6 Division of Neonatology, Department of Pediatrics, Saint-Luc University Hospital, UCLouvain, Brussels, Belgium; 7 Division of Neonatology, University & Polytechnic Hospital La Fe — Institute of Health Research La Fe (IIS La Fe), Valencia, Spain; 8 Neonatal Brain Group, Institut de Recerca Sant Joan de Déu (IRSJD), Barcelona, Spain; 9 Neonatology Service, Hospital Sant Joan de Déu Barcelona (SJD), and Department of Pediatrics, University of Barcelona (UB), Barcelona, Spain; 10 Department of Surgery and Medical-Surgical Specialties, Faculty of Medicine and Health Sciences, Universitat de Barcelona, Barcelona, Spain; 11 European Society for Paediatric Research, Satigny, Switzerland; 12 Department of Pediatrics, Dr. von Hauner Children’s Hospital, LMU University Hospital Munich, Munich, Germany; 13 German Center for Child and Adolescent Health, site Munich, Munich, Germany; 14 European Academy of Paediatrics, Brussels, Belgium; 15 Department of Neonatology, University Children’s Hospital Regensburg (KUNO), Hospital St. Hedwig of the Order of St. John of God, University of Regensburg, Regensburg, Germany

**Keywords:** Scientific community, Health services

**To the editors**,

We thank Drs Lakshminrusimha and Steinhorn for asking “Is it time to create a separate residency and department in neonatal critical care medicine?” [1], thereby sparking off a very timely discussion. We would like to add the European perspective, where Neonatology is now recognised as a designated Paediatric subspecialty in many countries and a formally accredited and widely endorsed Neonatology training framework, including neonatal critical care, already exists [2, 3].

In the European Union (EU), the Union Européenne des Médécins Spécialistes (UEMS) formally approves medical specialty-specific training. The “European Training Requirements” (ETR) are proposed by key stakeholders, usually specialists’ learned societies, who define the content of speciality-specific training and propose curricula. These then require the approval of the UEMS as the highest EU-specific accrediting organ.

The need for a unifying definition of neonatal specialist training throughout the EU member states has long been recognised and acted upon [4, 5]. In 1998, the European Academy of Paediatrics / European Board of Paediatrics supported the first ETR Neonatology, developed by the European Society for Paediatric Research (ESPR) [6]. The ETR Neonatology has since been updated twice, in 2007 and 2021.

With reference to the current debate [1], we note that the ETR Neonatology, Version 2021, contains many elements mentioned by Hill et al. as critical for comprehensive education and practice in Neonatology [7]. The ETR Neonatology, Version 2021, is furthermore officially endorsed by representatives of all 27 European National Neonatal Societies [8]. With this endorsement came two key mandates: first, to establish a board representing the interests of all European neonatologists, and second, to ensure access to accredited training programmes in neonatal medicine that provide the practical and theoretical foundation required to practise neonatology to the highest contemporary standards. The ESPR has fulfilled both mandates by (1) creating the European Board of Neonatal and Child Health Research (EBNCHR) which focusses on advocacy and research policy advances, and regularly updates the ETR Neonatology, and (2) expanding its widely subscribed catalogue of training programmes under the umbrella of the European School of Neonatology (ESN) [9]. This includes the academically oriented Master of Science in Neonatology (NOTE – Neonatal Online Training and Education, University of Southampton) and the practice-focused Master of Advanced Studies in Neonatology [9, 10]. Recently, the ESN was officially certified by the Foundation for International Business Administration Accreditation (FIBAA, Zurich, CH), receiving two distinctions: Certified Continuing Education Course and Excellence in Digital Education. Both programmes, NOTE and the ESN, deliver a standardised neonatal medicine curriculum for paediatricians pursuing sub-specialisation in neonatology, including neonatal critical care.

In 2025, Spain has recognised Neonatology as a subspecialty, thereby joining Germany, the UK, Poland and others where Neonatology and specific training requirements, including board certification, are well established. Thus, acknowledging the momentous efforts of the European Commission to close gaps and discrepancies in sub-specialty recognition and training between countries, and recognising the many complexities of establishing and implementing unified and accountable training schemes, we emphasise that greater transparency in neonatal training has now been achieved, at least for Europe.

Therefore, with the UEMS-endorsed neonatal training curriculum of the ESN in place, further progress has been made towards paving the way for pan-European standards for neonatology education. Concurring with Lakshminrusimha and Steinhorn’s call for better recognition of academic Neonatology, the ESPR and its organisational pillars, the ESN and EBNCHR, strive to facilitate this through international advocacy for research, innovation and education. Activities include international trainee mentoring programmes and specialist exchanges to nurture the next generation of neonatal physician scientists. And, importantly, giving access to a transparent, internationally consented curriculum and accreditation in Neonatology through the ESN.

In conclusion, Drs Lakshminrusimha and Steinhorn rightly emphasise the urgent need for universal recognition of neonatal critical care medicine. We support their urgent call for action to improve the recognition to retain staff and to enthuse medical graduates to consider a career in Neonatology. We think that a clear understanding of existing North-American and European frameworks will help inform global strategies and ultimately strengthen Neonatology worldwide.
